# G2LC: Resources Autoscaling for Real Time Bioinformatics Applications in IaaS

**DOI:** 10.1155/2015/549026

**Published:** 2015-10-04

**Authors:** Rongdong Hu, Guangming Liu, Jingfei Jiang, Lixin Wang

**Affiliations:** ^1^School of Computer, National University of Defense Technology, Changsha 410073, China; ^2^National Supercomputer Center, Tianjin 300457, China

## Abstract

Cloud computing has started to change the way how bioinformatics research is being carried out. Researchers who have taken advantage of this technology can process larger amounts of data and speed up scientific discovery. The variability in data volume results in variable computing requirements. Therefore, bioinformatics researchers are pursuing more reliable and efficient methods for conducting sequencing analyses. This paper proposes an automated resource provisioning method, G2LC, for bioinformatics applications in IaaS. It enables application to output the results in a real time manner. Its main purpose is to guarantee applications performance, while improving resource utilization. Real sequence searching data of BLAST is used to evaluate the effectiveness of G2LC. Experimental results show that G2LC guarantees the application performance, while resource is saved up to 20.14%.

## 1. Introduction

With significant advances in high-throughput sequencing technologies and consequently the exponential expansion of biological data, bioinformatics encounters difficulties in analysis of vast amounts of data. The need for storing and processing large-scale genome data, easy access to analyses tools, and efficient data sharing and retrieval has presented significant challenges. At present, cloud computing is a promising solution to address these challenges. Cloud computing offers near-infinite amount of resources capacity at a competitive rate and allows users to obtain resources on demand with pay-as-you-go pricing model. The elasticity and enormous capacity of cloud make it possible for bioinformatics applications to return results in a real time way. It will help speed up the bioinformatics research process and relieve the storage pressure of massive data.

IaaS (Infrastructure as a Service), as one important form of cloud computing, mainly leverages the virtualization technology to create multiple VMs (Virtual Machines) on a physical host and can support rapid deployment of large-scale applications [1]. Cloud providers can reduce power consumption by consolidating various applications into a fewer number of physical hosts and switching idle hosts to low-power modes. However, virtualization also creates a new problem. The application performance relies on effective management of VM capacity. One essential requirement of cloud computing is providing reliable QoS defined in terms of SLA (Service Level Agreements). SLA violation will bring economic penalties to cloud providers. Therefore, they always strive to ensure the agreed performance of individual VM. It is nontrivial because of the complexity of applications, various resources usage patterns, shared underlying hardware infrastructure, and the performance correlation and interference among applications.

The focus of this work is on the performance of bioinformatics applications in IaaS. This work tries to take advantage of cloud elasticity to deal with changes in application loads. A resource autoscaling method, G2LC, is proposed to provide suitable processing power for bioinformatics application, to keep up with the changes of sequence length. The method is based on statistical learning load forecasting algorithm. Application performance is guaranteed by adjusting the forecasted results, while minimizing resource usage. Real traces data of BLAST, one of the most widely used bioinformatics programs for sequence searching, is used to evaluate the effectiveness of G2LC. Experimental results show that G2LC can save more than 20% of the resources, while guaranteeing application performance.

The rest of this paper is organized as follows.  Section 2 describes the background.  Section 3 proposes the detailed design and implementation of G2LC and Section 4 presents the experimental evaluation.  Section 5 examines the related work and Section 6 makes the conclusions.

## 2. Background

According to [2], Real Time Systems (RTS) are those whose correctness depends not only on the logical results but also on the time in which such results are produced. In this type of applications, completion or response is always constrained by time. Failing in accomplishing this requirement could result in serious implications. Depending on the flexibility of such constraints, real time applications are generally classified into hard, firm, and soft. Hard real time applications are those where the nonfulfillment of the time constraints leads to system failure. Firm real time applications have hard constraints, but they allow certain level of tolerance. In the case of soft real time applications, the nonfulfillment of deadlines degrades the performance of system but does not destroy it by failure or crash. In this study, bioinformatics applications will be considered as soft real time applications. Regardless of the sequence length change, the result is required to return as quickly as possible.

Cloud computing is inherently real time and more specifically soft real time. Most existing cloud applications have stringent timing and performance requirements, such as voice and object recognition, image and video retrieval, financial systems, log processing, advertisement placement, and personalized recommendations. These applications are becoming increasingly latency sensitive and operating under demanding workloads that require fast response, for which some violations of the timing constraints are acceptable. For example, if an email system responds slowly, users may switch to another service provider. While the failure of the system to respond quickly may lead to customer churn and reduction on service provider's profits, it does not cause any catastrophic consequences.

On the other hand, cloud computing is also very suitable for RTS. In order to accomplish the time constraints, RTS normally demands large amount of computing resources. Cloud computing can offer this scalability. The virtualization and the resulting decoupling of infrastructure and application offered by cloud make it possible to rapidly scale the infrastructure to meet the resource requirements of the real time applications. Popular social applications, such as Facebook and Twitter, make further enhancement to enable real time communication. Major search engines jump in real time war by providing real time search results. Force.com provides real time integration with external cloud services such as Amazon Web Services, Facebook, Google App Engine, and Twitter.

However, the advent of other critical cloud computing targets such as the improvement of cost efficiency is creating a challenging atmosphere to real time applications. Cloud providers require not only accomplishing performance and time constraints of the applications, but also improving the resources utilization of data centers. The objective is to increase their profits while QoS is guaranteed. This balance is fundamental for the real time cloud.

## 3. Method: G2LC

### 3.1. VM Vertical Scaling

In IaaS, applications share the underlying hardware by running in isolated VMs. Each VM, during its initialization, is configured with a certain amount of resources (such as CPU, memory, and disk). A key factor for improving utilization efficiency is resource provisioning. The objective of VM vertical scaling is to ensure that VM capacity is matched with the workload, while overprovisioning wastes costly resources and underprovisioning degrades application performance. Existing virtualization technologies can adjust the capacity of a live VM locally based on time division multiplexing to maximize the resource utilization, also referred to as VM resizing.

Implementation of any policy is accompanied by operating costs. Chen et al. [3] set up a simulated environment and perform a preliminary experiment to illustrate the VM vertical scaling effect on three types of applications (CPU-, memory-, and network I/O-intensive). Experimental results show that the application performance degradation during the VM vertical scaling is smaller than that during VM migration, and the time of performance degradation is also shorter. The VM vertical scaling avoids the unnecessary VM migration by reallocating the spare resources to the heavy-loaded VM in very short time. By comparison, although VM migration can solve the performance problem, it spends much more time to do VM transmission, which generates significant interferences to the other colocated applications. Particularly, the network-intensive application receives serious interference when doing the VM migration, which is because much of the network I/O is preempted by the migration.

This work will adopt VM vertical scaling to adjust the VM processing capacity according to load changes of application. In IaaS virtualization platform, there are many mature tools (such as Xen (http://xenproject.org/), KVM (http://www.linux-kvm.org/page/Main_Page), and VMware (http://www.vmware.com/)) available for system manager to perform the monitoring and vertical scaling operations. For instance, we can use the Xen* xm* command to collect the CPU utilization of VM and use the Xen credit scheduler to set the CPU capacity limit of VM for vertical scaling.

### 3.2. Load Forecasting

One essential requirement of a real time cloud is providing reliable QoS defined in terms of SLA. The sequences processed by bioinformatics applications often have very different lengths causing dynamic resources usage pattern. The consolidation of VMs can lead to performance degradation when an application encounters an increasing demand resulting in an unexpected rise of resources usage. This may lead to SLA violation—increasing response time. Overprovisioning may help to ensure SLA, but it leads to inefficiency when the load decreases. The optimal strategy is to timely adjust resources provisioning according to the actual demands of the application. One precondition of this approach is to find out the future load.

In this work, we will adopt KSwSVR, proposed in our previous work, as our load prediction method [4]. It is based on statistical learning technology which is suitable for the complex and dynamic characteristics of the cloud computing environment. KSwSVR integrates an improved SVR (Support Vector Regression) algorithm and Kalman smoothing technology and does not require access to the internal details of application. The improved SVR gives more weight to more important data than standard SVR, using the historical information more reasonably. Kalman Smoother is employed to eliminate the noise of resources usage data coming from measurement error. In comparison with AR (Autoregression), BPNN (Back Propagation Neural Networks), and standard SVR, KSwSVR always has the minimum prediction error facing every type of resources and different predicted steps.

### 3.3. G2LC

We propose* G2LC* to improve the load forecasting-based resource autoscaling method with two adjustment mechanisms—*global gain* for predicted value and* local compensation* for error.

*(i) SLA Definition*. Resource utilization is commonly used by data center operators as a proxy for application performance because of the monotonic relationship between them and the fact that utilization is easily measurable at the OS level. In-depth researches on this relationship were also conducted, such as in [5, 6]. As it lies outside the sphere of our work, without loss of generality, we also adopt resource utilization to indicate QoS. In this study, SLA model of application is defined as follows:

(1) 1i−1∑j=1i−1Fxjalloc>xjuse=cslaVi≤slaVFy=0,if  y  is  true1,if  y  is  false.

*x*_*j*_^alloc^ and *x*_*j*_^use^ separately represent actual resources allocation value and real resources usage of application in time interval *j*. cslaV_*i*_ is the average SLA violation rate before interval *i*. It is the indication of the average QoS for VM and is restrained by slaV which is confirmed after the negotiation between cloud service providers and customers. slaV represents the user's tolerance for performance degradation. It is usually smaller than 5% for real time applications. As long as VM resource utilization is below 100%, that is, *x*_*j*_^alloc^ > *x*_*j*_^use^, we judge that the resource is enough and there is no SLA violation.

Typically, users rent a VM with a certain capacity from IaaS providers. The resource configuration of the VM is unchanged at run time. This is currently a common practice, but it cannot effectively deal with the changing load. We take it as a comparison object in this work, and the resource saving rate is calculated as follows:

(2) $$\begin{array}{c} \text{resource saving rate}=\frac{\sum_{i=1}^{T}\left(x_{\text{Fixed},i}-x_{\text{G2LC},i}^{\text{alloc}}\right)}{\sum_{i=1}^{T}x_{\text{Fixed},i}}. \end{array}$$

**Figure 1:**
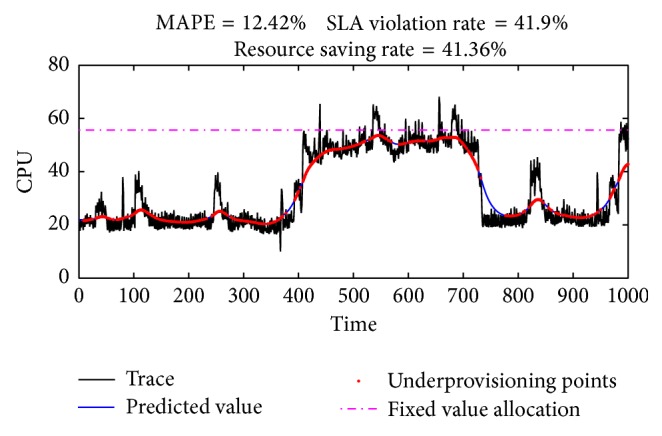
Resource autoscaling directly using predicted value.

*(ii) Global Gain for Predicted Value.* Even if the load prediction algorithm has high prediction accuracy, can we directly use the predicted value as the ultimate resource supply? We randomly select a piece of data from Google trace (https://code.google.com/p/googleclusterdata/) to analyze this issue. The data is linearly converted before test. Test results are shown in Figure 1.

If the resource allocated to VM is a fixed value, the CPU capacity must be more than 55.65 (horizontal dotted line) to keep the SLA violation rate below 5%. It can be seen from the figure that the loads continue to fluctuate, and the prediction accuracy of KSwSVR is acceptable. The MAPE (Mean Absolute Percentage Error) is only 12.42%. Compared with the fixed value allocation, the method based on the predicted value saves 41.36% of the resource usage in total. But the result of using the predicted value directly for the resource allocation is up to 41.9% SLA violation (red dots, underprovisioning points) rate, which is clearly unacceptable.

The root cause of this problem is the prediction error that any prediction algorithm cannot avoid. When the prediction target is variable, the error will be more notable. Therefore, we need to adjust the predicted value when making the VM scaling scheme.

**Figure 2:**
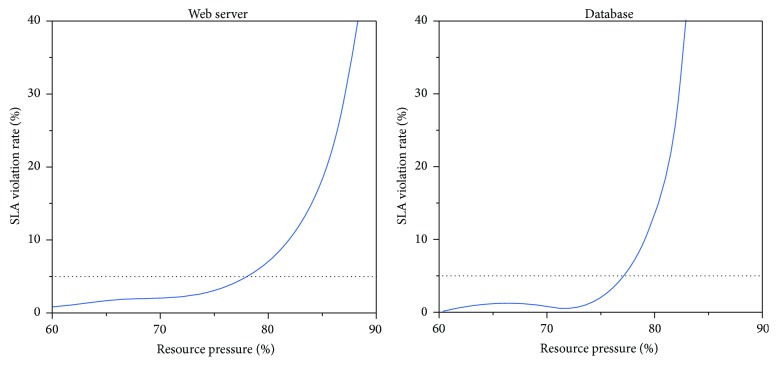
Relationship between application performance and resource pressure.

Researchers from North Carolina State University, NetApp, and Google have studied the relationship between application performance and resource pressure (ratio of the total resource demand to the total resource allocation) [7]. They tested a web server and a database server. The result is shown in Figure 2. When the resource utilization of server exceeds 80%, application performance will seriously decline. If the target is to ensure the SLA violation rate less than 5%, the resource utilization of web servers and database servers must be kept, respectively, at 78% and 77% or less. The main reason is that current level of technology cannot effectively deal with load fluctuations. We need to provide a certain amount of redundant resources to maintain the application performance.

Inspired by this, we intend to adjust the forecast result $\hat{x_{t}}$ by adding a gain coefficient *C*_*g*_ > 1, using more resources to meet the needs of real time applications:

(3) $$\begin{array}{c} x_{t}^{\text{alloc}}=C_{g}\hat{x_{t}}. \end{array}$$

**Figure 3:**
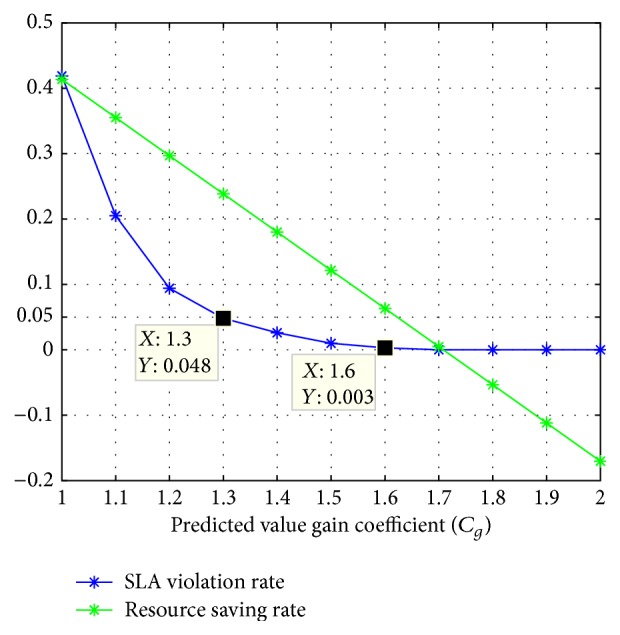
Impact of *C*_*g*_ on application performance and resource usage.

We need to address the overprovisioning and underprovisioning problems in determining the amount of this part of redundant resources. For this purpose, we use an incremental traversal method to test the impact of the value of *C*_*g*_ on application performance and resource usage. The result is shown in Figure 3.

In the experiment, the value range of *C*_*g*_ is set to [1, 2]. In other words, the resource allocation amount increases from predicted value to its double. As *C*_*g*_ increases, more resources are added to application, and SLA violation rate decreases rapidly (*SLA violation rate*); that is, application performance is quickly enhanced. When *C*_*g*_ = 1.3, SLA violation rate drops below 5%. When *C*_*g*_ > 1.6, there is no SLA violation event. On the other hand, when *C*_*g*_ increases, the resource consumed by application also increases, and the cost advantage, relative to fixed value allocation, decreases linearly (*resource saving rate*). When *C*_*g*_ increases to 1.7,* resource saving rate* is reduced to zero; that is, the total resources usage of prediction-based dynamic allocation is quite equal to the one of fixed value allocation. If we continue to increase *C*_*g*_,* resource saving rate* will become negative, and dynamic resource scaling will waste more resources.

If we only consider the resource utilization, the smaller the value of *C*_*g*_, the better the management effect. However, to meet the application performance requirements, *C*_*g*_ should be set to 1.3 for the load in the experiment. That is, the resource utilization should be maintained at about 77% ≈ 1/1.3. It is consistent with the conclusion of [7] cited before. We also randomly selected a number of other load data from Google to test. The results showed that 1.3 is an ideal gain coefficient.

**Figure 4:**
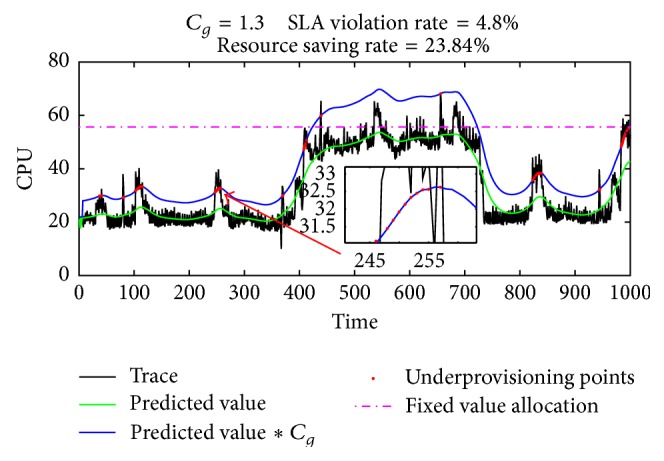
Resource autoscaling with gain coefficient *C*_*g*_.

*(iii) Local Compensation for Error*. If the gain coefficient *C*_*g*_ is set to 1.3, the result of resource autoscaling based on load forecasting is shown in Figure 4.

As can be seen from the figure, after adding the gain coefficient *C*_*g*_, the overall performance of the application is guaranteed well. Most of the time, because of the introduction of the gain coefficient, there is a significant gap between the resource provisioning curve and trace curve. This gap represents the wasted resource. SLA violation event mainly concentrated near the peak load, as it is difficult for prediction algorithm to deal with the temporary change of object. It is a problem that all current prediction algorithms cannot solve well. Therefore, predicted value needs postprocessing before being used. If we want to further improve the resource utilization, and also to ensure meeting application performance requirements, we need to amend the locality where the SLA violation events happen.

To this end, we further improve the resource scaling method and introduce a local error compensation mechanism to deal with the concentrated underprovisioning. The purpose is to reduce the SLA violation events as much as possible, providing space for further improving the resource utilization (by reducing gain coefficient *C*_*g*_).

It should be noted that, in practice, IaaS service providers can perceive the VM underproviding based on VM resource utilization, but they cannot learn the specific deficiency. Therefore, we cannot directly use the difference between trace data and predicted value as the error compensation (which is actually the optimal solution).

**Figure 5:**
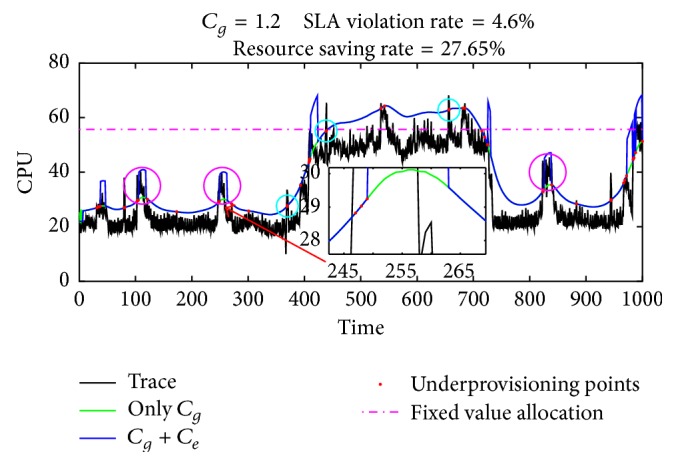
Resource autoscaling with *C*_*g*_ and *C*_*e*_.

We continue to introduce a local error compensation coefficient *C*_*e*_ based on (3):

(4) xtalloc=CeCgxt^Ce=αcardv−Trd⁡V=i ∣ xt−iuse=xt−ialloc,  i∈1,2,…,we,

where *w*_*e*_ is the windows width; that is, error compensation mechanism will take into account the resource usage of the past *w*_*e*_ periods to develop the resource scaling scheme of the next period. Because the VM resource usage cannot exceed the amount of its total resource, *x*_*t*−*i*_^use^ = *x*_*t*−*i*_^alloc^ means lack of resource. *V* is a set of SLA violation events occurring in last *w*_*e*_ periods. card(*V*) denotes the elements number of set *V*. *T*_rd⁡_ is a threshold value. Once the number of SLA violation events within the window exceeds the threshold, the error compensation mechanism will be triggered. *α* = 1.1 is a constant; that is, error compensation amount increases at a rate of 10% each time.  Figure 5 shows the dynamic resource autoscaling process under the combinational effect of global gain coefficient *C*_*g*_ and local error compensation coefficient *C*_*e*_.

On the whole, compared with Figure 4, SLA violation rate reduces from 4.8% to 4.6%. Not only is the application performance improved slightly, but also more resource is saved.* Resource saving rate* (compared with fixed value allocation) increases from 23.84% (only *C*_*g*_) to 27.65% (*C*_*g*_ + *C*_*e*_). That is, the introduction of reasonable local error compensation mechanism creates space for reducing the gain coefficient *C*_*g*_, while improving application performance and resource utilization.

Particularly, the most intuitive change generated by the reduction of *C*_*g*_ from 1.3 to 1.2 is the shrink of the gap between the resource provisioning curve and trace curve. In other words, less resource is wasted. Another significant change is that the resource provisioning curve is no longer as smooth as before. The introduction of local error compensation coefficient *C*_*e*_ and window *w*_*e*_ makes the resource management system respond rapidly to the load spikes (as shown in magenta circles). In addition, we introduce *T*_rd⁡_ as a resource compensation mechanism trigger condition threshold, mainly to avoid the unnecessary compensation operation triggered by glitches (transient load peaks, as shown in cyan circles). It helps to improve resource utilization and enhances the system stability.

Another significant change is that although the number of SLA violation events (marked as red dots) does not reduce much, their distribution has changed a lot. In Figure 4, the SLA violation events concentrated near the peak load. In Figure 5, the red dots become decentralized. For end-user applications, they may encounter sporadic request response delay but will not suffer long time “fake system halt.” This will help to improve the user experience.

**Figure 6:**
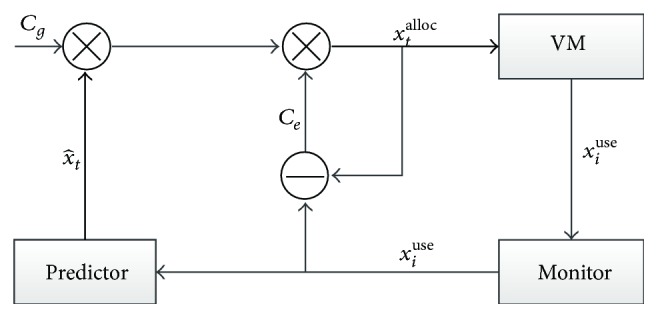
Control process of G2LC.

So far, we have adjusted the predicted values at two levels: global gain and local compensation. The control process of resources autoscaling is shown in Figure 6. Monitor collects VM resources utilization data *x*_*i*_^use^ and sends them to the predictor. Predictor predicts the resource consumption $\hat{x_{t}}$ in the next control cycle. Finally, the resource scaling scheme *x*_*t*_^alloc^ is figured out after the adjusting of global gain *C*_*g*_ and local compensation *C*_*e*_.

## 4. Experiment with BLAST

Focusing on CPU utilization is a good way to understand the application performance, as it is typically proportional to the end-user productivity. Thus, CPU utilization can support greater transparency between cloud service providers and customers. Measuring and reporting CPU utilization is also a simple, affordable, and adequate way of gauging data center efficiency. Most importantly, many of the existing bioinformatics applications are compute-intensive applications. Hence, in this work, we focus on the CPU utilization of application.

It should be noted that the experiments in this paper mainly focus on CPU. So, the experimental conclusions surely apply to CPU-intensive applications. However, G2LC is also applicable to other types of applications (such as the memory-/disk-/network-intensive ones), because the existing virtualization technology can dynamically split these types of resources in a fine-grained way and the forecasting algorithm also applies to these resource objects.

### 4.1. Experiment Setup

BLAST (Basic Local Alignment Search Tool) [8] is one of the most widely used bioinformatics programs for sequence searching. It addresses a fundamental problem in bioinformatics research. BLAST is an algorithm for comparing primary biological sequence information, such as the amino-acid sequences of different proteins or the nucleotides of DNA sequences. A BLAST search enables a researcher to compare a query sequence with a library or database of sequences and identify library sequences that resemble the query sequence above a certain threshold. The heuristic algorithm it uses is much faster than other approaches, such as calculating an optimal alignment. This emphasis on speed is vital for making the algorithm practical on the huge genome databases currently available.

The effectiveness of G2LC is evaluated by using open real-world BLAST workload traces (http://ammatsun.acis.ufl.edu/amwiki/index.php/Prediction) rather than historical data generated by ourselves for the purpose of giving comparable and reproducible results.

The owners of the traces have comparatively assessed the suitability of several machine learning techniques for predicting spatiotemporal utilization of resources by BLAST [9]. They also extended Predicting Query Runtime (PQR) to the regression problem. BLAST was executed against the nonredundant (NR) protein sequence database from NCBI (National Center of Biotechnology Information). Given an input sequence, BLAST searches a database for similar sequences and calculates the best alignment of the matched sequences. Single nucleotide sequences of varying lengths served as input in the search process.

Different from their work focusing on run time prediction, this study is to guarantee the external performance of bioinformatics applications—returning the results in real time for different size of sequences (load). The traces provide the real search time of each sequence in nonvirtualization environments. If the search time of a sequence is longer, we believe it will need more computing resources in real time environment. Therefore, in the experiment, the search time attribute of sequence in traces is used as the load input. We expect to achieve a real time output by dynamic resource scaling.

### 4.2. Experimental Results

**Figure 7:**
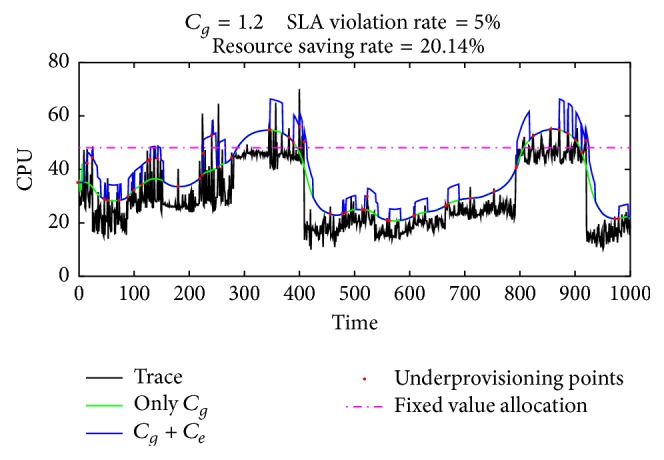
G2LC on BLAST trace.

Experimental results are shown in Figure 7. With the change in the length of the sequence, the processing power of VM must be kept up with this change if we require BLAST to output the search result in a real time model. With the same parameters setting as before, G2LC not only guarantees the overall performance of BLAST, in this context, but also tries to minimize the gap between the resource provisioning curve and trace curve. The overall SLA violation rate is maintained at 5%. Compared with fixed value allocation with the same QoS, G2LC saved up to 20.14% of the resources.

**Figure 8:**
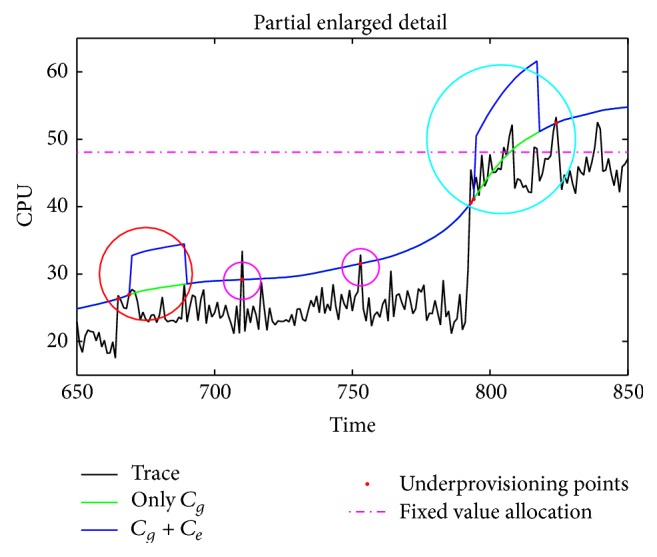
G2LC effect in detail.

To further analyze the effect of G2LC, we extract a small portion of the data to be described in detail, as shown in Figure 8. The global gain coefficient *C*_*g*_ makes the resource provisioning curve generally above the trace curve, guaranteeing the average performance of BLAST around the acceptable range. The introduction of local error compensation coefficient *C*_*e*_ makes the G2LC respond rapidly to the peak load growth (as shown in cyan circle). On the basis of *C*_*g*_, *C*_*e*_ further reduces the probability of SLA violation event. In addition, the trigger threshold of resource compensation mechanism, *T*_rd⁡_, avoids the unnecessary compensation operation at spikes (as shown in magenta circles). It helps to save resource and enhances the system stability. But in some cases, *T*_rd⁡_ and window *w*_*e*_ will lead to a slight negative impact. As shown in red circle, once the number of SLA violation events in the window exceeds the threshold *T*_rd⁡_, the local compensation mechanism is triggered, regardless of whether the subsequent load increases. If subsequent load did not continue to increase, this will lead to a waste of resources. But its impact on the global effect is very slight, because the duration of resource waste cannot exceed the window width *w*_*e*_.

## 5. Related Work

In this section, we briefly review some recent approaches on building and running bioinformatics applications on cloud platform.

As the field of bioinformatics expands, some researches have utilized cloud computing to deliver large computing capacity and on-demand scalability. Crossbow [10] is a cloud enabled tool that combines the aligner Bowtie and the SNP caller SOAPsnp and uses Hadoop for parallel computing. Rainbow [11] is a cloud-based software package that can assist in the automation of large-scale whole-genome sequencing (WGS) data analyses. It copies input datasets to Amazon S3 and utilizes Amazon's computing capabilities to run WGS data analyses pipelines. CloudMap [12] is a pipeline that greatly simplifies the analysis of mutant genome sequences from raw FASTQ reads to mapping plots and short lists of candidate mutations. CloudBurst [13] is a parallel read-mapping algorithm used for next-generation sequence data of the human genome and other reference genomes. It is implemented on a Hadoop-based cluster and aims to optimize the parallel execution. RSD-Cloud [14] runs a comparative genomics algorithm on Amazon EC2 for ortholog calculations across a wide selection of fully sequenced genomes. These projects focus on the solutions of specific problems by developing a tool or method.

Cloud BioLinux [15] is one of the early attempts to simplify the deployment and execution of bioinformatics applications on the cloud. It is a VM configured for high-performance bioinformatics using cloud platforms. At the beginning, over 135 bioinformatics tools have been deployed and configured on the VM. Li et al. [16] presented Hadoop-based applications employed in bioinformatics, covering next-generation sequencing and other biological domains. They described how to obtain an increase in performance by utilizing Hadoop on a cloud computing service and explored different alignment tools and applications that perform sequence alignment. Widera and Krasnogor [17] used Google App Engine computing platform as the computing resource. They introduced the method of building the computer generated protein models used in the protein structure prediction. The proposed Protein Models Comparator is their solution to the problem of large-scale model comparison and can be scaled for different data sizes. Hung and Hua [18] combined two different heterogeneous architectures, software architecture-Hadoop framework and hardware architecture-GPU, to develop a high performance cloud computing service, called Cloud-BLASTP, for protein sequence alignment. Cloud-BLASTP takes advantage of high performance, availability, reliability, and scalability. Cloud-BLASTP guarantees that all submitted jobs are properly completed, even when running job on an individual node or mapper experience failure.

Liu et al. [19] introduced a novel utility accrual scheduling algorithm for real time cloud computing services. The real time tasks are scheduled nonpreemptively with the objective to maximize the total utility. Two different time utility functions were proposed to model the real time applications for cloud computing that need not only to reward the early completions but also to penalize the abortions or deadline misses of real time tasks. Kim et al. [20] investigated power-aware provisioning of VMs for real time services. They modeled a real time service as a real time VM request and provisioned VMs using DVFS scheme. Several schemes were proposed to reduce power consumption by hard real time services and power-aware profitable provisioning of soft real time services.

In comparison to all these studies, G2LC is a general solution for bioinformatics applications to improve resource utilization in IaaS. It does not require access to the internal details of application and executes autoscaling scheme only based on the analysis of application resource utilization data. The purpose is to reduce service costs, while ensuring QoS at the same time.

## 6. Conclusions

With the rapid growth of next-generation sequencing technologies, more and more data have been discovered and published. To analyze such huge data, the computational performance becomes an important issue. The main focus of this work is on the performance of bioinformatics applications in IaaS. We try to take advantage of cloud elasticity to deal with changes in application loads, making it able to return a result in real time way. A resource autoscaling method, G2LC, is proposed to provide the right amount of resources, to keep up with the changes of sequence length. A statistical learning-based algorithm is adopted for load forecasting. While minimizing resource usage, application performance is guaranteed by adjusting the forecasted results with global gain and local error compensation. Real BLAST trace data is used to evaluate the effectiveness of G2LC. Experimental results show that G2LC can save more than 20% of the resources, while guaranteeing application performance.
